# The effect of transcutaneous electrical nerve stimulation-assisted therapy combined with pregabalin in the treatment of trigeminal neuralgia

**DOI:** 10.12669/pjms.41.6.11988

**Published:** 2025-06

**Authors:** Kan Yue, Yinsheng Chen, Shengrong Xu, Ruilin He, Zongbin Jiang

**Affiliations:** 1 Kan Yue Department of Pain Management, The Second Affiliated Hospital of Guangxi Medical University, Nanning, Guangxi Province 530007, P.R. China; 2 Yinsheng Chen Shanghai East Hospital Clinical Medical College, Nanjing Medical University, Shanghai, 200120, P.R. China; 3 Shengrong Xu Department of Pain Management, The Second Affiliated Hospital of Guangxi Medical University, Nanning, Guangxi Province 530007, P.R. China; 4 Ruilin He Department of Pain Management, The Second Affiliated Hospital of Guangxi Medical University, Nanning, Guangxi Province 530007, P.R. China; 5 Zongbin Jiang Department of Pain Management, The Second Affiliated Hospital of Guangxi Medical University, Nanning, Guangxi Province 530007, P.R. China

**Keywords:** Effect, Pregabalin, Transcutaneous electrical nerve stimulation, Trigeminal neuralgia

## Abstract

**Objective:**

Transcutaneous electrical nerve stimulation (TENS) therapy has become an emerging option for acute and chronic pain. This study aimed to investigate the efficacy of TENS combined with pregabalin in treating trigeminal neuralgia (TN).

**Methods:**

Clinical records of TN patients treated with pregabalin in the Department of Pain Management, the Second Affiliated Hospital of Guangxi Medical University, from October 2022 to October 2024 were retrospectively analyzed. Patients were divided into the TENS group and the non-TENS group based on the treatment method. The pain severity, sleep quality, levels of pain-inducing factors, quality of life, and incidence of adverse reactions in the two groups were compared before and after four weeks of treatment.

**Results:**

Records of 205 patients were included in the analysis, with 104 in the TENS group and 101 in the non-TENS group. After four weeks of treatment, the visual analog scale (VAS) and The Pittsburgh Sleep Quality Index (PSQI) scores in the TENS group were significantly lower than those in the non-TENS group (all P<0.05). The levels of pain-inducing factors and improvement in quality of life in the TENS group were considerably better compared to the non-TENS group (all P<0.05). There was no significant intergroup difference in the incidence of adverse reactions (P>0.05).

**Conclusions:**

In TN patients, a combination of TENS and pregabalin was associated with higher treatment efficiency without an increase in adverse reactions compared to pregabalin monotherapy.

## INTRODUCTION

Trigeminal neuralgia (TN) is characterized by intense, transient, sudden, and recurrent facial pain that significantly impacts patients’ quality of life.^1^–^3^ The first-line treatment of TN includes antiepileptic drugs such as carbamazepine.^4^ However, this method is associated with high incidence of drug resistance and numerous side effects such as blurred vision and dizziness.^1^,^4^,^5^ Therefore, identifying new, safer treatment options for TN is crucial. Pregabalin is a novel gabapentinoid with analgesic properties that binds to the alpha-2-delta subunit of the voltage-gated Ca2+ channels.^6^ Studies show that pregabalin is effective in reducing pain levels, but its effect is dose-dependent and not suitable for long-term use.^7^,^8^

Recently, transcutaneous electrical nerve stimulation (TENS), which uses mild electrical current, has emerged as a promising method of pain relief for patients with TN.^9^ Studies have showed that the low-voltage current efficiently dilates blood vessels, increases blood flow, and reduces inflammation, improving patients’ clinical symptoms.^9^,^10^ However, while previous research has demonstrated the effectiveness of combined TENS/pregabalin in patients with postherpetic neuralgia and neuropathic pain after stroke,^11^,^12^ there are currently no reports on the efficiency of TENS + pregabalin regimen for TN. This study aimed to analyze the effectiveness and safety of TENS combined with pregabalin to provide new clinical direction for more efficient treatment of TN.

## METHODS

Clinical records of all TN patients who have received pregabalin at the Department of Pain Management, the Second Affiliated Hospital of Guangxi Medical University, between October 2021 and October 2024 were retrospectively selected. Patients were retrospectively divided into a TENS group and a non-TENS group based on whether they received TENS adjuvant therapy during the use of pregabalin. This research was approved by the hospital ethics committee. The retrospective design of this study exempted patients from the requirement of informed consent. In order to protect privacy, all patient information has been anonymous. Doctors’ clinical decisions are grouped according to patients’ requirements, clinical symptoms and drug treatment effects. In order to ensure comparability, the baseline characteristics of the two groups were statistically analyzed before the test. All outcome data were collected before treatment and one month after treatment. This retrospective cohort study did not use randomization or blinding for patients and researchers.

### Ethical Approval:

The ethical committee of the Second Affiliated Hospital of Guangxi Medical University approved this study with the number: 2024-KY-(0767), Date: December 2^nd^, 2024.

### Inclusion criteria:

- Meets the TN diagnostic criteria.^1^
- Visual, pain, and sensory abnormalities with obvious distribution of neural innervation areas;
- Age ≥ 18 years old.
- Not taking analgesic medication within one month before receiving pregabalin treatment.
- Complete clinical data.

### Exclusion criteria:

- Patients with epilepsy, head injuries, severe heart disease.
- Patients with secondary TN (such as multiple sclerosis, space-occupying lesions, etc.).
- Other factors can cause facial pain in patients.
- Female patients during lactation and pregnancy.

### Pregabalin:

Oral Pregabalin (manufacturer: Pfizer, USA) was administered at the initial dosage of 75 mg/time twice a day. If there were no adverse reactions after three days of treatment, the dosage was increased to 150 mg/time, twice a day, for four weeks.

### TENS:

The equipment selected was a CEFALY instrument from STV Med (Belgium), with 4 cm × 4 cm silicone rubber electrodes coated with conductive adhesive. The anode was placed at the tender point, and the trigeminal nerve branch pathway and the cathode was placed in the corresponding area. The frequency was set to 2-100 Hz, and the current intensity was set to 0.5-1 mA. The specific parameters were adjusted according to the patient’s reaction. Following these criteria for TENS equipment parameter adjustment: The current intensity starts at 0.5 mA and gradually increases to 1 mA depending on patient tolerance; Pulse width is 200μs, and frequency is modified based on patient suffering. Chronic individuals use 2-10 Hz, while acute pain patients need 80-100 Hz. Based on patient feedback, the parameters were adjusted to cause slight tingling in the pain location without discomfort. Care was taken to ensure that the current was applied to the whole pain area without obvious discomfort to the patient. The electrode parameters were adjusted multiple times based on the patient’s response to the treatment to make the patient feel a tingling sensation in the painful area, suppress the pain, and make the patient feel comfortable. The treatment was carried out once a day, 20 minutes per time, five times a week, for a total of four weeks of treatment.

### Baseline data and related indicators were collected before and after four weeks of treatment and included:

- Pain level evaluated based on the Visual Analog Scale (VAS), with a score range of 0-10 points, and the lower score indicating lighter pain sensation.
- Sleep quality evaluated based on the Pittsburgh Sleep Quality Index (PSQI), with a score range of 0-21 points. The lower score correlates with, better sleep quality.
- Serum levels of pain-inducing factors, including β-endorphin (β-EP), substance P (SP), and 5-hydroxytryptophan (5-HT), measured by radioimmunoassay using the reagent kit from Beijing Furui, China.
- Quality of life, assessed according to the World Health Organization Quality of Life Abbreviated (WHO-QOL-BRAF), including physical health, environmental factors, psychological status, and social function. The score range for each dimension was 0-100 points, with higher scores indicating better quality of life.
- Incidence of adverse reactions, including fatigue, hypotension, diarrhea, and dizziness.
- The primary result of this study was the alteration in VAS score pre- and post-treatment. Secondary outcome markers encompass: 1) Alterations in PSQI score; 2) Modifications in serum pain-inducing variables (β-EP, SP, and 5-HT); 3) Variations in quality of life score (WHO-QOL-BRAF); 4) Frequency of adverse events.

### Statistical analysis:

All data were analyzed using SPSS 25.0 (IBM Corporation, Armonk, NY, USA) and GraphPad Prism 8.0 (GraphPad Software, Inc.). Evaluate the normality of the distribution of quantitative data using the Shapiro - Wilk test. Normal distribution data were represented by mean ± standard deviation, an independent sample t-test was used for inter-group comparison, and a paired t-test was used for intra-group comparison before and after the treatment. Non-normally distributed data were represented by median and interquartile ranges. Mann Whitney U test is used for inter-group comparisons, and Wilcoxon signed-rank test was used for intra-group comparisons. The count data were represented by the number of cases, using the chi-square test or Fisher’s exact probability method. When *P<0.05*, the difference was considered statistically significant. This study only included patients with complete clinical data, therefore no data was missing. Our inclusion criteria require patients to have comprehensive clinical data, including all evaluation indexes before and after treatment.

Because this study is retrospective, the sample size was not determined. The posterior statistical power analysis was carried out. In order to detect the clinical significance difference of one point by using the main outcome index VAS score with a standard deviation of 1.5, when α = 0.05 and β = 0.20, 95 patients were needed in each group. This criterion is satisfied by our sample size of 104 TENS cases and 101 non-TENS cases.

## RESULTS

This retrospective study included records of 205 eligible patients, 110 males and 95 females. The median age was 48 (42-56) years. There were 104 patients in the TENS group and 101 patients in the non-TENS group, with no significant difference in the baseline clinical characteristics such as age, gender, affected nerves, affected side, body mass index (BMI), and underlying diseases between the two groups of patients (all *P*>0.05) (Table-I).

**Table-I:** Comparison of Basic Clinical Characteristics between Two Groups.

Characteristics	TENS group (n=104)	Non TENS group (n=101)	Z/t/χ^2^	P
Age (years), M(P25/P75)	48.5 (42.5-57)	47 (42-54)	-1.027	0.304
Male, n (%)	51 (49.0)	59 (58.4)	1.812	0.178
BMI (kg/m^2^), mean±SD	22.9±2.9	23.7±3.6	-1.800	0.073
***Affected nerves, n (%)***				
Ⅱ	24 (23.0)	16 (15.9)	3.903	0.142
Ⅲ	35 (33.7)	47 (46.5)		
Ⅱ+Ⅲ	45 (43.3)	38 (37.6)		
***Affected side, n (%)***				
Left	51 (49.0)	47 (46.5)	0.129	0.720
Right	53 (51.0)	54 (53.5)		
Coronary heart disease, n (%)	18 (17.3)	16 (15.8)	0.080	0.778
Diabetes, n (%)	19 (18.3)	15 (14.9)	0.433	0.511
Hypertension, n (%)	20 (19.2)	16 (15.8)	0.407	0.524

Data are present as mean ± standard deviation (SD) or median and interquartile range (IQR) or number (%). Transcutaneous electrical nerve stimulation (TENS); Body Mass Index (BMI).

**Fig.1:**
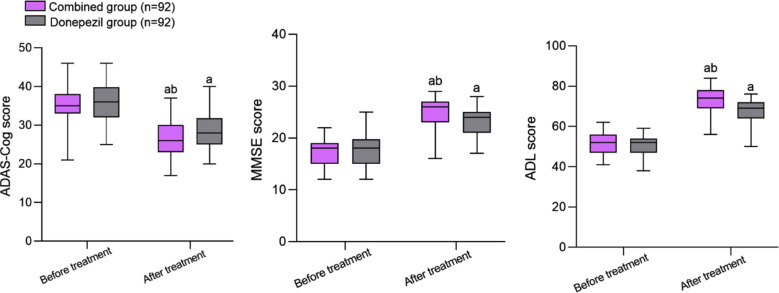
Comparison of VAS score and PSQI score between Two Groups. T0: before treatment; T1: after treatment. Transcutaneous electrical nerve stimulation (TENS); visual analogical scale (VAS); Pittsburgh sleep quality index (PSQI).

Before treatment, the two groups had no significant difference in VAS and PSQI scores (all *P*>0.05). After treatment, the VAS and PSQI scores of both groups significantly decreased compared and were significantly lower in the TENS group compared to the non-TENS group (all *P*<0.05) (Fig.1).

**Fig.2:**
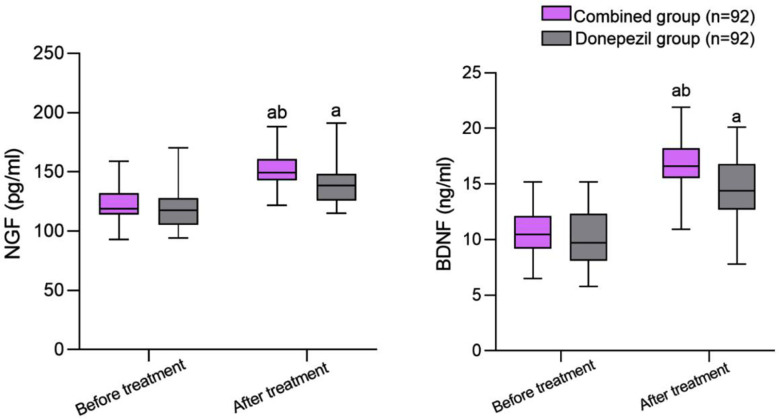
Comparison of β-EP, SP, and 5-HT levels between two groups. T0: before treatment; T1: after treatment. Transcutaneous electrical nerve stimulation (TENS); β-endorphin (β-EP); Substance P (SP); 5-hydroxytryptamine (5-HT).

Pretreatment levels of β-EP, SP, and 5-HT were comparable in the two groups (all *P*>0.05). After treatment, the levels of β-EP and 5-HT in both groups increased, while the level of SP decreased significantly compared to before treatment (all *P*<0.05). Post-treatment levels of pain-inducing factors in the TENS group were considerably better than those in the non-TENS group (all *P*<0.05) (Fig.2).

**Fig.3:**
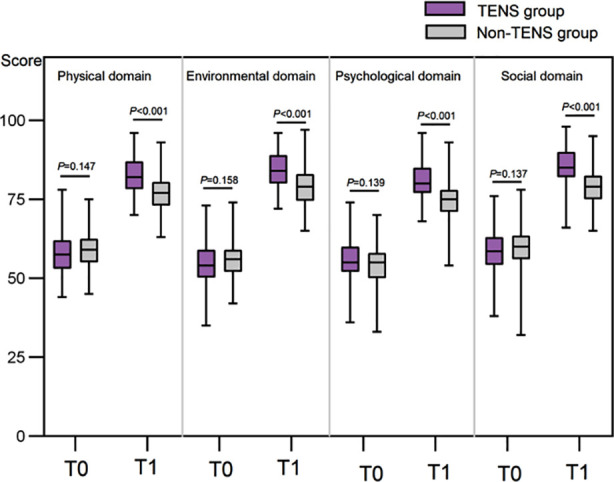
Comparison of WHO-QOL-BRAF scores between two groups. T0: before treatment; T1: after treatment. Transcutaneous electrical nerve stimulation (TENS).

Before treatment, the two groups had no significant differences in physical health, environmental factors, psychological status, and social function scores (all *P*>0.05). Post-treatment scores of the physical, environmental, psychological, and social domains in both groups significantly increased and were considerably higher in the TENS group than in the non-TENS group (all *P*<0.05) (Fig.3).

Several adverse events were reported in the TENS group, including one case of fatigue, two cases of diarrhea, and one case of dizziness, with an incidence of approximately 4.0% (4/104). In the non-TENS group, there were two cases of fatigue, one case of hypotension, two cases of diarrhea, and two cases of dizziness. The incidence of adverse events was approximately 7.0% (7/101). There was no significant difference in the incidence of adverse events between the two groups (*P*>0.05) (Table-II).

**Table-II:** Comparison of Adverse Reactions between Two Groups.

Group	n	Fatigue	Hypotension	Diarrhea	Dizziness	Total incidence rate
TENS group	104	1 (1.0)	0 (0.0)	2 (2.0)	1 (1.0)	4 (4.0)
Non TENS group	101	2 (2.0)	1 (1.0)	2 (2.0)	2 (2.0)	7 (7.0)
*χ^2^*						0.906
*P*						0.503^b^

b represents Fisher’s exact test calculation.

## DISCUSSION

This study investigated the efficacy and safety of TENS combined with pregabalin in treating TN. The results demonstrate that the combined treatment can significantly reduce the pain and levels of pain-inducing factors in TN patients and improve their sleep quality and quality of life without an increase in adverse reactions. This study confirms that TENS combined with pregabalin is associated with higher benefits to TN patients. Combining the two, thus, produces a synergistic effect, thereby improving the effectiveness of treatment.

The results from this study indicate that the concurrent application of TENS and pregabalin produces a synergistic effect, enhancing the therapeutic outcomes across multiple dimensions. And this study showed that in TN patients, the combined treatment was associated with significantly improved VAS and PSQI scores compared to the pregabalin monotherapy. This indicates that TENS-assisted pregabalin treatment can significantly reduce patient pain and improve sleep. This is consistent with the studies of Barbarisi et al.^11^ and Xu et al.^13^ Pregabalin mainly exerts its effects through mechanisms such as regulating neurotransmitter release and inhibiting neuronal overexcitation, while TENS directly acts on nerve conduction pathways and interferes with the transmission of pain signals.^7^–^14^ Therefore, it is plausible that the combined treatment can exert synergistic effects at different stages, effectively reducing pain perception and improving sleep quality severely affected by pain.^11^–^14^

TENS combined with pregabalin may have many benefits. TENS activates the descending pain suppression system by stimulating large afferent nerve fibers, which leads to opioid release and β-EP increase.^12^-^14^ This study showed that while both treatment regimens significantly improved the levels of pain-inducing factors β-EP, 5-HT, and SP, the effect was considerably stronger in patients who received the combined treatment. We may speculate that this effect is due to pregabalin’s function as a calcium channel blocker. The reduced influx of calcium ions and decreased release of neurotransmitters such as norepinephrine and SP subsequently reduce pain excitability and sensation.^7^,^8^,^15^ At the same time, TENS can promote local blood circulation, increase the supply of oxygen and nutrients, stimulate nerve endings, inhibit nerve conduction, and reduce the transmission of pain signals.^13^,^14^,^16^ Zhou et al.^16^ used TENS during the perioperative period of chest surgery and demonstrated an increase in the plasma β-EP levels. The research also suggests that TENS and drug therapy can increase 5-HT levels in patients.^17^

This study further confirms these observations. TN patients suffer from long-term pain, limited physical function, increased psychological pressure, and reduced social interaction, leading to a serious decline in their quality of life.^18^,^19^ This study used WHO-QOL-BRAF to evaluate the changes in the quality of life of TN patients before and after treatment. The results showed that after treatment, the scores of various dimensions of quality of life in both groups of patients increased compared to before treatment, and were considerably higher in the TENS group. These results further confirm the superiority of the combined TENS-pregabalin treatment in improving the quality of life of TN patients. Reducing pain is the key to improving quality of life. As the degree of pain decreases, the patient’s physical condition improves, allowing them to better participate in daily activities.^20^,^21^ Pain relief also helps to improve the psychological state of patients, alleviate negative emotions such as anxiety and depression, and enhance their social functioning, which, in turn, reduces the economic burden caused by disease.^19^

Previous studies have showed that pregabalin treatment is associated with certain side effects, such as headache, dizziness, and drowsiness.^22^ This study did not detect a significant difference in the incidence of adverse effects between the two groups, confirming the safety of the combined approach. A randomized controlled trial by Li et al.^23^ showed that electroacupuncture combined with carbamazepine, a voltage-dependent Na(+) channel blocker, considerably reduces the incidence of adverse events in TN patients. The discrepancy between this study’s results and precious observations may be related to the relatively small sample size of the current cohort, further strengthening the need for future larger-scale studies. The therapeutic effect depends on individualising TENS parameters: acute pain requires high frequency (80-100 Hz) and chronic pain requires low frequency (2-10 Hz). Older patients need lower current intensity (0.5-0.7 mA) and younger patients higher (0.8-1 Ma). Adjust the electrode location according to the nerve branches (eye, maxillary, mandibular); Treatment can be lowered from 5 times a week to 2-3 maintenance treatments.”

This study has important clinical implications. It confirms that TENS-assisted pregabalin treatment for TN can significantly alleviate pain levels and improve sleep quality and quality of life. The conclusion of this study also paves the way for developing new approaches to treating TN. However, it is important to note that the effectiveness of TENS may vary due to individual differences, device parameters, and treatment time. Therefore, further high-quality research is needed to better meet patients’ needs, improve treatment outcomes, and develop effective personalized treatment plans.

### Limitations:

Firstly, it is a single-center retrospective study with a small sample size and a certain risk of selection bias. Secondly, The median age of patients is 48 years old, which is younger than that described in the literature (60-70 years old), which may restrict the application of the results to older patients because young people may accept and respond better to TENS. Thirdly, without long-term follow-up, the long-term effectiveness of TENS treatment cannot be determined. Additionally, longer follow-ups are needed to monitor for new adverse reactions or a decline in treatment effectiveness over time. Fourthly, due to individual differences, the strength of TENS cannot be standardized. Therefore, higher-quality research is needed in the future to confirm the effectiveness and safety of TENS.

## CONCLUSION

Transcutaneous nerve electrical stimulation and pregabalin for trigeminal neuralgia reduce pain, improve sleep, and improve life quality. Based on these data, we advocate TENS as an adjuvant to pregabalin, especially for individuals with low drug tolerance or poor monotherapy response. Research with bigger samples is needed to prove long-term effectiveness and create personalized methods.

### Authors’ contributions:

**KY and YC:** Study design, literature search and manuscript writing.

**KY, YC, SX, RH and ZJ:** Data collection, data analysis and interpretation. Critical review.

**KY:** Manuscript revision and validation, critical analysis.

All authors have read, approved the final manuscript and are accountable for the integrity of the study.
